# Pulse corticosteroid therapy in the treatment of steroid-refractory immune checkpoint inhibitor-related pneumonitis: Case report and review

**DOI:** 10.3389/fimmu.2022.994064

**Published:** 2022-08-31

**Authors:** Kuan-Chang Lai, Yi-Han Hsiao, San-Chi Chen

**Affiliations:** ^1^ Department of Medicine, Taipei Veterans General Hospital, Taipei, Taiwan; ^2^ Department of Chest Medicine, Taipei Veterans General Hospital, Taipei, Taiwan; ^3^ Division of Medical Oncology, Center for Immuno-oncology, Department of Oncology, Taipei Veterans General Hospital, Taipei, Taiwan

**Keywords:** Pulse corticosteroid therapy, steroid-refractory, immune checkpoint inhibitors, pneumonitis, immune-related adverse event, nivolumab

## Abstract

Immune checkpoint inhibitors (ICIs) have demonstrated promising therapeutic outcomes in treating a variety of malignancies, but immune-related adverse events (irAE) may develop. Among all the irAE, immune-related pneumonitis was relatively common and life-threatening. High-dose corticosteroid was recommended for the initial management, but a part of patients developed steroid-refractory pneumonitis. Other immunosuppressants were recommended, but the optimal treatment is still controversial. Here, we report two cases of steroid-refractory immune-related pneumonitis who were successfully treated with pulse corticosteroid therapy. Case 1 was hepatocellular carcinoma treated with nivolumab for 5 months. She developed acute respiratory distress syndrome due to grade 4 immune-related pneumonitis that was refractory to intravenous methylprednisolone 2 mg/kg/day treatment. Methylprednisolone 500 mg for 3 days followed by 2 mg/kg/day steroid as maintenance therapy was given. Subsequently, her pneumonitis was regressed, and the endotracheal tube was successfully removed on day 9 after the start of pulse therapy. Case 2 presented with grade 4 immune-related pneumonitis in spite the use of methylprednisolone 1 mg/kg for his skin rash. Pulse corticosteroid therapy was prescribed, then his pneumonitis was completely regressed on day 12. In this report, we demonstrated the potential role of pulse corticosteroid therapy for steroid-refractory pneumonitis.

## Introduction

In recent years, immune checkpoint inhibitors (ICIs) including anti-PD-1/PD-L1 and anti-CTLA-4 have played an important role in the treatment of various types of cancer. ICIs augment antitumor immunity by enhancing priming of CD4 T cells and cytotoxic function of CD8 T cells, which demonstrated a durable response in a part of cancer patients. However, with the activation of the immune system, ICIs also increased the risk of autoimmune toxic effects, termed immune-related adverse events (irAE). The common sites develop irAE including skin, lung, liver, and endocrine system.

Among all the irAE, immune-related pneumonitis is relatively common and life-threatening. The incidence of immune-related pneumonitis is about 5%, and the mortality rate of grade 3–4 immune-related pneumonitis is approximately 25%. The time to develop is approximately 9 days to19.2 months after the start of ICI therapy. Immune-related pneumonitis has various clinical manifestations, ranging from no symptoms, fever, shortness of breath, and even to respiratory failure and death. The immune-related pneumonitis is a diagnosis of exclusion. Pulmonary edema, infection, radiation pneumonitis, and cancer progression should be considered in the differential diagnosis. The radiological features of pneumonitis in chest computed tomography imaging could be various, including ground glass opacities, cryptogenic organizing pneumonia pattern, interstitial change pattern, consolidation, and traction bronchiectasis. Current guidelines recommend high-dose (1–4 mg/kg) prednisolone/equivalent corticosteroids for the initial management of immune-related pneumonitis. The efficacy of high-dose corticosteroid therapy reached approximately 70%–80% of response rate (1).

In some patients, pneumonitis could not be resolved despite high-dose corticosteroids for 48 to 72 h, which is known as steroid-refractory irAE. According to the current guidelines, other immunosuppressive agents, such as TNF-α inhibitor, intravenous immunoglobulin, cyclosporine, mycophenolate mofetil (MMF), cyclophosphamide, and tocilizumab, should be considered for severe or steroid-refractory irAE. Balaji et al. reported that 12 patients with steroid-refractory pneumonitis were treated with a TNF-α inhibitor (infliximab), intravenous immunoglobulin, or combined therapy, but the death rate was up to 75% (2). Beattie et al. described 26 patients with steroid-refractory pneumonitis treated with infliximab, MMF, or both, but only 10 patients (38.5%) had clinical improvement (3). Therefore, the optimal treatment of steroid-refractory pneumonitis is still controversial.

Here, we firstly reported two cases of steroid-refractory pneumonitis who were treated with pulse corticosteroid successfully.

## Case report

### Case 1

**Figure 1:**
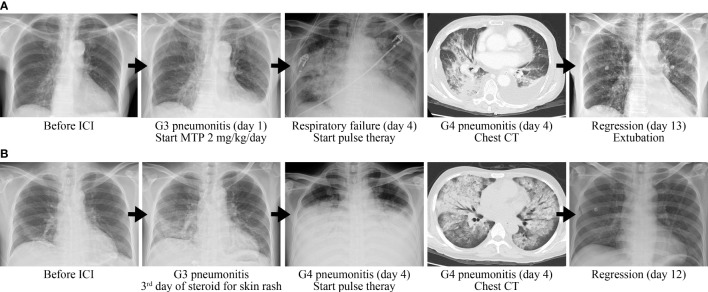
Immune-related pneumonitis. Case 1 was presented in **(A)**, and case 2 in **(B)**.

A 77-year-old woman had a medical history of hypertension and hepatitis B. She had never drunk alcohol, smoked tobacco, or taken illicit drugs. There was no previous medical history of lung disease and autoimmune disease. She was diagnosed with stage IB hepatocellular carcinoma (HCC) and then underwent resection in 2019. Due to repeated recurrence, she received several times of trans-arterial chemoembolization. Lenvatinib was prescribed but with poor response. Nivolumab was given for second-line treatment with the best response of stable disease. After 5 months of treatment, she complained of progressive exertional dyspnea. The oxygen saturation was down to 93%, and arterial blood gas analysis revealed paO_2_ 58 mmHg while the patient was breathing ambient air. Physical examination revealed bilateral rale breathing sounds. The electrocardiography disclosed a sinus rhythm without significant ST segment or T-wave change. The laboratory data disclosed WBC 4,900/µl, neutrophil-to-lymphocyte ratio 3.78, CRP 1.46 mg/dl, troponin I within the normal reference range, NT-proBNP 1,494 pg/ml, and d-dimer 5.6 µg/ml. Chest computed tomography demonstrated bilateral diffuse ground grass opacities but no evidence of pulmonary embolism. Other than that, irAE involving other organs was not observed. The impression was grade 3 immune-checkpoint inhibitor-related pneumonitis so that intravenous methylprednisolone 2 mg/kg/day was given. However, she presented with progressive hypoxia with a PaO_2_/FiO_2_ ratio of 135 which met the Berlin criteria of acute respiratory distress syndrome. Due to respiratory failure, she received endotracheal intubation with mechanical ventilation support. Repeat chest CT showed progressive bilateral crazy paving and consolidation. After consulting the pulmonologist, the patient was treated with intravenous pulse methylprednisolone 500 mg for 3 days followed by 2 mg/kg/day of steroid as maintenance therapy. In the following days, hypoxemia was rapidly improved (PaO_2_/FiO_2_ ratio 410) and chest X-ray image showed regression of bilateral lung infiltration. The endotracheal tube was successfully removed on day 9 after the start of pulse therapy, and she was discharged within 14 days **(** **Figure 1A** **)**.

### Case 2

A 44-year-old man had a medical history of stage IV chronic kidney disease. He did not smoke tobacco nor drink alcohol. No previous medical history of lung disease and autoimmune disease was documented. He was diagnosed with metastatic HCC in in June 2021 and received lenvatinib as first-line target therapy with poor response. Subsequently, nivolumab was prescribed as the second-line therapy. However, 10 h after nivolumab infusion, the patient experienced generalized pruritus and fever. The physical examination revealed a body temperature of 38.5°C and disseminated maculopapular skin rashes. There was no significant change in complete blood count/differential count and biochemistry data. Due to suspicion of intraabdominal infection, empirical antibiotics with ceftazidime was given. Grade 2 checkpoint inhibitor immune-related skin toxicity was also impressed, so intravenous methylprednisolone 1 mg/kg/day was prescribed. Three days later, the fever and rash alleviated but the patient developed dry cough and progressed dyspnea. The oxygen saturation was down to 67%, and the PaO_2_/FiO_2_ ratio was 66.3 under a non-rebreathing mask. The laboratory data showed WBC 11,800/µl, neutrophil-to-lymphocyte ratio 27.16, and CRP 21.92 mg/dl. Chest CT showed bilateral, multifocal ground grass lesions and no evidence of pulmonary embolism. Grade 4 immune-related pneumonitis was impressed. Since pneumonitis is refractory to the dose of methylprednisolone 1 mg/kg/day for 3 days, pulse corticosteroid therapy (methylprednisolone 500 mg/day for 3 days) was prescribed followed by 2 mg/kg/day methylprednisolone and mycophenolate mofetil (1,000 mg twice a day) for maintenance. The pneumonitis was regressive, and hypoxemia was improved (PaO_2_/FiO_2_ ratio 437.5). The patient no longer needed oxygen therapy 8 days from the start of pulse corticosteroid therapy **(** **Figure 1B** **)**.

## Discussion

Corticosteroids were widely used immunosuppressive agents. They inhibit a broad spectrum of immune cells and the synthesis of numerous inflammatory cytokines. At present, there is no clear definition for pulse corticosteroid therapy. In general, doses above 250 mg prednisone or equivalent steroids for days are considered as pulse corticosteroid therapy. The pulse steroid therapy plays a role in life-threatening autoimmune diseases, such as Goodpasture disease, necrotizing glomerulonephritis, and severe lupus nephritis. The clinical use of pulse corticosteroid therapy is summarized in **Table 1**.

**Table 1:** The role of pulse corticosteroid therapy.

Research	Disease	Cases	Treatment	Outcome
**Autoimmune disease**				
2020, Condon et al. (4)	Lupus nephritis	50	• Two doses of rituximab (1 g), MTP (500 mg) on days 1 and 15, and maintenance treatment of MMF	• 1 year of complete remission rate: 52%
2021, Mejía-Vilet et al. (5)	Lupus nephritis	362	• MTP 500 mg for 3 days	24-week overall response rate • Multitarget treatment group: 83.5%; • Cyclophosphamide group: 63%
**Immune-related adverse events**				
2021, Taliansky et al. (6)	Encephalitis	11	• Low-dose steroids: 1 patient • Pulse MTP 1 g/day for 5 days: 10 patients (1 patient: +plasma exchange, 1 patient: + plasma exchange and cyclophosphamide)	Neurological improvement rate: • Low-dose steroids: 0% (0/1) • Pulse therapy: 90% (9/10)
2017, Makarious et al. (7)	Myasthenia gravis	23	• Pulse MTP: 5 patients	• Mortality rate: 40%
2020, Manohar et al. (8)	Acute interstitial nephritis	14	• Prednisolone (0.5-1 mg/kg/day) for 7 patients • Pulse MTP therapy 250-9750 mg for 7 patients	2-month complete response rate • Prednisolone: 60% • Pulse MTP therapy: 71.4%
2021, Oleas et al. (9)	Acute interstitial nephritis	8	• Prednisone 1 mg/kg/day: 5 patients • Pulse MTP 250-500 mg for 3 days: 3 patients	• 3-month complete response rate: 87% • Pulse therapy: 66.7%
2017, Ozaki et al. (10)	Cystitis	1	• MTP 500 mg for 3 days	• Complete recovery
**Steroid-refractory ICI-related pneumonitis**				
2021, Balaji et al. (2)	Immune pneumonitis	12	• Infliximab, IVIG, or combination therapy	• Death rate: 67%
2021, Beattie et al. (3)	Immune pneumonitis	26	• TNF inhibitor, MMF, cyclophosphamide, or combination therapy	• Recovery rate: 38%
2020, Utsumi et al. (11)	Immune pneumonitis	1	• Pulse MTP 1,000 mg for 3 days, tacrolimus, cyclophosphamide 500 mg therapy for 1 day	• Recovery

MMF, mycophenolate mofetil; MTP, methylprednisolone; IVIG, intravenous immunoglobulin.

Pulse corticosteroid therapy could rapidly achieve an immunosuppressive effect and avoid long-term steroid exposure in autoimmune diseases. A prospective lupus nephritis study reported 50 patients with class III, IV, or V lupus nephritis receiving rituximab, MMF, and two additional doses of 500 mg intravenous methylprednisolone. After 1 year of follow-up, the complete remission rate was 52% (4). A randomized controlled trial reported treatment strategies for lupus nephritis, which compared the efficacy of the intravenous cyclophosphamide group and combination therapy group (calcineurin inhibitor, MMF, and glucocorticoid). All the patients received 500 mg daily intravenous methylprednisolone as pulse therapy for 3 successive days and 0.6 mg/kg oral prednisone as maintenance treatment. After 6 months, the overall response rate in both groups was higher than 60% (5). A systematic review demonstrated that in comparison to the oral steroid-treated or untreated group, intravenous pulse corticosteroid therapy did not increase the risk of adverse effects, including neuropsychiatric, metabolic effect, and infectious complications (12). Therefore, intravenous pulse corticosteroid therapy could be considered a safe option for life-threatening autoimmune diseases.

To our best knowledge, the use of pulse steroid therapy for irAE was only reported in a few case series and case reports. In a report with 11 immune-related encephalitis cases, pulse steroid therapy demonstrated a high neurological improvement rate of 90% (6). Therefore, pulse steroid therapy was recommended for the initial treatment of immune-related encephalitis in the guideline (1). A case–cohort study enrolled 14 patients who were tissue-proven or were clinically suspicious of immune-related acute interstitial nephritis. Some patients were treated with variable doses of pulse corticosteroid therapy from 250 to 9750 mg. The complete response rate was 71.4% which is higher than in those treated without pulse therapy (8). Another study described eight patients who were diagnosed with immune-related acute interstitial nephritis which was confirmed by pathology. Three patients underwent intravenous pulse corticosteroid therapy. Renal function was recovered in two of them (67%), but one patient developed chronic kidney disease (9). A clinical case report demonstrated a man with advanced-stage lung squamous cell carcinoma who experienced grade 3 cystitis caused by nivolumab. However, supportive care and symptomatic management could not alleviate the symptoms. After receiving consecutive intravenous 500-mg doses of methylprednisolone for 3 days and oral prednisolone as maintenance treatment, the cystitis was improved (10). Besides, in a study of immune-related myasthenia gravis, five patients treated with pulse steroid therapy resulted with a myasthenia gravis-relevant mortality of 40% [2020 Huang]. For immune-related pneumonitis, only a case report described a man with metastatic non-small cell lung cancer who experienced steroid-refractory pneumonitis with pulse corticosteroid therapy failure. Subsequently, the pneumonitis was improved with the combination of high-dose corticosteroids, tacrolimus, and cyclophosphamide pulse therapy (11). Taken together, the role of pulse corticosteroid therapy for the treatment of irAE is potential but still lacks strong evidence for pneumonitis.

## Conclusion

The current recommendation of the steroid-refractory immune pneumonitis is still controversial. The optimal treatment strategy presents a great challenge to clinicians. While pulse corticosteroid therapy offers a cost-effective treatment option, more data are needed to support the role of pulse corticosteroid for steroid-refractory pneumonitis.

## Data availability statement

The original contributions presented in the study are included in the article/supplementary material. Further inquiries can be directed to the corresponding author.

## Ethics statement

This study was reviewed and approved by institutional review board of Taipei Veterans General Hospital (TPEVGH IRB No.: 2021-08-005AC). The patients/participants provided their written informed consent to participate in this study. Written informed consent was obtained from the individual(s) for the publication of any potentially identifiable images or data included in this article.

## Author contributions

Analyzed the data: K-CL, Y-HH, S-CC. Contributed reagents/materials/analysis tools: Y-HH, S-CC. Contributed to the writing of the manuscript: K-CL, S-CC. All authors contributed to the article and approved the submitted version.

## Funding

This study is supported by Taipei Veterans General Hospital (V111C-131).

## Acknowledgments

The authors wish to acknowledge the support of Taipei Veterans General Hospital (V111C-131).

## Conflict of interest

The authors declare that the research was conducted in the absence of any commercial or financial relationships that could be construed as a potential conflict of interest.

## Publisher’s note

All claims expressed in this article are solely those of the authors and do not necessarily represent those of their affiliated organizations, or those of the publisher, the editors and the reviewers. Any product that may be evaluated in this article, or claim that may be made by its manufacturer, is not guaranteed or endorsed by the publisher.

## Abbreviations

ICIs, immune checkpoint inhibitors; irAE, immune-related adverse events; MMF, mycophenolate mofetil; HCC, hepatocellular carcinoma; TACE, trans-arterial chemoembolization; CNI, calcineurin inhibitor.
